# Back-wall cystic figures are not undoubtedly pathognomonic of benignity: malignant and infectious mimickers in thyroid lesions

**DOI:** 10.1530/ETJ-26-0070

**Published:** 2026-04-22

**Authors:** Pierre-Yves Marcy, Juliette Thariat, Marc Tassart

**Affiliations:** ^1^ Department of Radiodiagnostics and Interventional Radiology; IMASUD Radiology Group, PolyClinics ELSAN MediPole Sud, Clinique les Fleurs, Quartier Quiez, Ollioules, France; ^2^ Department of Radiation Oncology, François-Baclesse Comprehensive Cancer Center, Caen, France; ^3^ Laboratoire de Physique Corpusculaire, IN2P3, CNRS, UMR 6534, Caen, France; ^4^ Service de Radiologie Diagnostique et Interventionnelle, CHU TENON, Est Parisien, Paris, France

Dear Editor,

We read with great interest the article by Marchand *et al.* recently published in ETJ, entitled ‘Can back-wall cystic figures of thyroid nodules predict benignity?’ (1). The authors present prospective data suggesting that back-wall cystic figures (BWCFs) reduce the probability of malignancy within EU-TIRADS 4 thyroid nodules, approaching that of EU-TIRADS 3 nodules, thus supporting the reduction of unnecessary fine-needle aspiration cytology (FNAC). Their work is methodologically sound and clinically stimulating; we congratulate the authors and would like to offer some additional comments.

While the authors draw attention to a well-known sonographic sign, it is crucial to emphasize that the ‘back-wall cystic figures’ are fundamentally a phenomenon of relative acoustic attenuation and sound transmission rather than a direct histological biomarker. Posterior acoustic enhancement occurs when a lesion attenuates the ultrasound beam less than the surrounding thyroid parenchyma, allowing relatively greater sound transmission and stronger echoes from deeper tissues. Rare purely cystic lesions, colloid cysts, and more often hemorrhagic cysts, spongiform nodules, and cystic transformation within thyroid adenomas exemplify this due to their near absence of internal scattering. However, attenuation characteristics alone are insufficient to definitively establish nodule benignity; malignant and non-neoplastic conditions may show similar appearances (Fig. 1).

First, certain thyroid cancers, including cystic variants of papillary thyroid carcinoma (PTC), may exhibit prominent cystic change and posterior enhancement. Although purely cystic nodules are overwhelmingly benign, cystic transformation in PTC is well documented, occurring in approximately 2–18% of cases (2). These EU-TIRADS 4 lesions often display mural nodules, internal septations, and occasionally back-wall cystic figures. Recent studies indicate that malignancy risk in partially cystic nodules is primarily driven by the morphology of the solid component; Shi *et al.*’s meta-analysis reported that eccentric configuration of the solid portion and microcalcifications significantly increase malignancy risk (3). Notably, microcalcifications can sometimes be difficult to distinguish from echogenic colloid granules, which are often interpreted as benign comet-tail artifacts associated with back-wall cystic figures. Histologically, malignant cystic PTC may contain trapped colloid within neoplastic epithelial clusters (2). Therefore, the presence of internal echogenic foci with posterior enhancement does not formally exclude carcinoma.

Moreover, cystic metastatic lymph nodes from papillary microcarcinomas of the thyroid isthmus, particularly in the central neck compartment, may exhibit striking posterior enhancement due to homogeneous intranodal necrosis and can be misdiagnosed as benign thyroglossal duct cysts (TDCs) without careful anatomical and imaging evaluation. Moreover, approximately one percent of TDC cases may give rise to papillary primary cancer, arising from adjacent thyroid tissue. Therefore, BWCF EU-TIRADS 4 nodules located along the midline of the neck should be interpreted with extreme caution (4).

**Figure 1:**
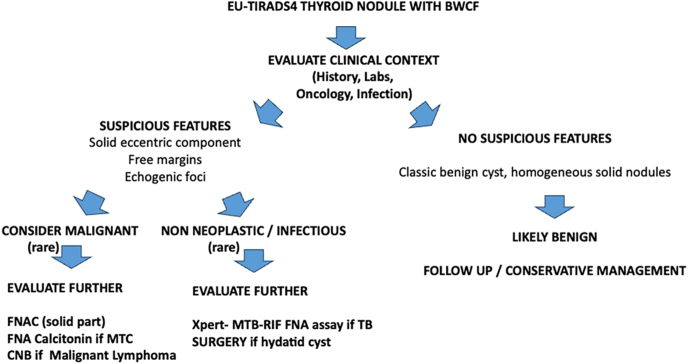
Management of EU-TIRADS 4 thyroid nodule with BWCF. FNAC, fine needle aspiration cytology; CNB, core needle biopsy; TB, tuberculosis; MTC, medullary thyroid carcinoma.

Medullary thyroid carcinomas (MTCs) also may occasionally display cystic-like features due to intratumoral necrosis or hemorrhage, which can lead to false-negative FNAC (accuracy <60%), highlighting the value of *in situ* FNA calcitonin measurement (5).

Second, primary thyroid MALT lymphoma may present with low internal attenuation due to high cellularity and relative paucity of fibrous stroma, producing posterior enhancement in up to 92% of cases (6). Particular attention should be paid to the reticular pattern and cervical lymphadenopathies, especially in the setting of chronic lymphocytic thyroiditis, which can complicate accurate assessment (7).

High-grade tumors, including primary malignant aggressive lymphoma and primary or metastatic thyroid sarcomas, may outgrow their vascular supply, resulting in central necrosis and cystic-appearing components with enhanced sound transmission. The resulting intratumoral fluid or hemorrhagic degeneration produces anechoic or hypoechoic regions with posterior enhancement, reinforcing that cystic appearance does not preclude malignancy (8).

Third, rare non-neoplastic entities should also be considered. Thyroid tuberculosis (TTB) is exceedingly rare, reported in 0.1–0.003% of postmortem studies, in 0.2% of chronic thyroiditis specimens, and in up to 14% of miliary tuberculosis cases. Interestingly, Bulbuloglu *et al.* reported that TTB presented as the only extrapulmonary manifestation in 87% of the cases, and as thyroid cysts in 2.6% of those cases. Thyroid tuberculosis may produce caseous necrosis and cyst-like sonographic appearances with posterior enhancement (9). The Xpert MTB/RIF assay enables the detection of Mycobacterium tuberculosis and rifampicin resistance from FNAC samples.

Primary thyroid hydatidosis, although exceptional (<1% of hydatid cases), poses diagnostic and therapeutic challenges. According to Gharbi’s classification, type IV hydatid-related thyroid cysts may present as hypoechoic heterogeneous lesions without daughter vesicles or detached membranes, but with back-wall cystic figures, often misleading on ultrasound examination. This is worthy to note that fine-needle aspiration is absolutely contraindicated due to the risk of spillage, dissemination, and anaphylaxis (10).

In conclusion, BWCFs in EU-TIRADS thyroid nodules are generally considered almost pathognomonic of thyroid benignity; however, BWCFs primarily reflect acoustic attenuation and necrotic fluid transformation rather than benign histology *per se*. This underscores the importance of carefully considering the patient’s clinical, biological, and infectious context. It is also crucial to emphasize that FNAC is formally contraindicated in rare instances of misidentified thyroid hydatid disease.

## Declaration of interest

The authors declare that there is no conflict of interest that could be perceived as prejudicing the impartiality of the work reported.

## Funding

This work did not receive any specific grant from any funding agency in the public, commercial, or not-for-profit sector.
